# Tunable Nonlinear Optical Response of ITO Films with Au@Ag Bimetallic Nanoparticles

**DOI:** 10.3390/nano13101631

**Published:** 2023-05-13

**Authors:** Tingzhen Yan, Ruijin Hong, Jiqing Lian, Chunxian Tao, Hui Lin, Qi Wang, Zhaoxia Han, Dawei Zhang

**Affiliations:** 1 Engineering Research Center of Optical Instrument and System, Ministry of Education and Shanghai Key Lab of Modern Optical System, University of Shanghai for Science and Technology, Shanghai 200093, China; tzyancn@163.com (T.Y.); cxtao@live.cn (C.T.); linh8112@163.com (H.L.); shelly3030@163.com (Q.W.); zxhan@usst.edu.cn (Z.H.); dwzhang@usst.edu.cn (D.Z.); 2 Department of Printing and Pack Aging Engineering, Shanghai Publishing and Printing College, No. 100 Shuifeng Road, Shanghai 200093, China; lianjiqing1990@163.com

**Keywords:** ITO films, Au@Ag colloid, nonlinear saturation absorption, localized surface plasmon resonance, epsilon-near-zero

## Abstract

The nonlinear optical (NLO) response of indium tin oxide films covered with Au@Ag colloid layer was characterized by a femtosecond single-beam open aperture (OA) Z-scan technique in this study. As the Au@Ag thickness increased, the transition from saturated absorption (SA) to reverse saturated absorption (RSA) was found in these ITO matrix composites. The nonlinear absorption coefficient for these composite materials can be regulated from −6.85 × 10^−7^ m/W to 26.06 × 10^−7^ m/W. In addition, this work also characterized the structure, morphology, and other optical properties of the specimen, and the finite-difference time-domain (FDTD) results were consistent with the experimental results. The NLO response of the ITO/Au@Ag composites can be attributed to the phase properties, synergistic competition effect, strong interaction based on the epsilon-near-zero (ENZ) mode, and localized surface plasmon resonance (LSPR) between the indium tin oxide films and Au@Ag.

## 1. Introduction

Nonlinear optics play a crucial role in modern functional devices and systems for photonics. The nonlinear optical phenomena have a wide range of applications in optical communication, all-optical information processing and storage, spectral technology, quantum information technology, Q-modulated lasers, and other fields [1]. The widespread use of nonlinear optics highlights the importance of nonlinear optical materials. However, the media typically exhibit weak optical nonlinearities under the action of strong coherent light [2]. Enhancing the nonlinear optical (NLO) response of materials [3,4] and realizing their applications in ultrafast switching, information processing, and on-chip nonlinear devices have received a great deal of research attention in the last decade. To further enhance the intrinsic weak NLO properties, the multifunctionality and practicality of the media should be improved to meet their targeted applications. Saturable absorbers are the only way to excite passively modulated Q lasers, and scientists are quite interested in the diversity and selectivity of saturable absorber materials. However, current saturable absorber materials still face many challenges in the development of pulsed lasers, such as the low modulation depth of graphene, the low stability of black phosphorus, and the low nonlinear effects of other materials. So far, scientists believe that saturable absorbers should have higher nonlinear effects, higher nonlinear refractive indices, lower unsaturated losses, and other advantages. In addition, significant efforts have been made to develop an ideal optical limiter in the last few decades. RSA, multiphoton absorption, nonlinear scattering, etc., are the major nonlinear mechanisms behind this limiting action. Therefore, it is essential to study the third-order nonlinear optical properties of these materials. To solve the above problems, researchers have developed hybrid semiconductors with metallic nanostructures [5,6,7,8], which can not only combine the respective advantages of metals and semiconductors but also obtain excellent adjustable optoelectronic performance by simple modifications [8].

Recently, transparent conductive oxides (TCOs) have attracted particular attention because they can achieve intriguingly large NLO responses in the frequency range where the dielectric tends to be close to zero. Indium tin oxide films (N-type oxide semiconductors, a commonly used TCO, one of the most common epsilon-near-zero (ENZ) materials), have extremely strong NLO responses in their ENZ region [9]. Since then, there have been numerous studies on the NLO response of ITO matrix materials within its ENZ region. In 2021, Ma et al. [10] proposed the principle method to synchronously modulate the saturated absorption (SA) and reverse saturated absorption (RSA) of ITO films, which greatly broadened their ability to modulate the nonlinear optical response in the ENZ region. In 2022, Lau et al. [11] showed significant saturable absorption characteristics of ITO films prepared by applying lower oxygen partial pressures and then used them as saturable absorbers to achieve Q-tuned pulse outputs in the 1 μm band and 1.5 μm. The unique linear and NLO properties of ENZ structures provide a new way of thinking about integrated optical device design.

Noble metal nanoparticles exhibit unique localized surface plasmon resonance (LSPR) absorption in the visible spectrum region and can be modulated from the UV to the NIR by varying parameters, such as size, structure, shape, and surrounding dielectric environment [12,13]. The localized electric field enhancement effect can significantly improve the material properties of the medium and can be used in studies such as surface-enhanced Raman spectroscopy and nonlinear optics [14]. Shiju E. et al. [15] synthesized Au@Ag core–shell nanostructures using a chemical method, exhibiting RSA behavior, and the nonlinear absorption enhanced the Ag thickness. The good NLO response of the Au@Ag structure ensures its potential for future NLO applications. The combination of different materials will give it some new synergistic properties and show different characteristics. In this paper, we constructed a hybrid ENZ-metal (ITO/Au@Ag) structure through a very simple manufacturing process, achieving tunable NLO properties for ITO matrix composites. By controlling the thickness of Au@Ag, the NLO response of the composite materials showed a transition from SA to RSA. Nonlinear absorption also appeared to be enhanced to some extent. In addition, the effects of Au@Ag on the linear optics, morphology, and ENZ properties of the ITO films are discussed. The electronic field distribution of ITO films covered with Au@Ag colloids was calculated by using finite-difference time-domain (FDTD). The experimental results provided reliable theoretical support for the application of ITO bimetallic nanoparticles in NLO optical devices.

## 2. Methods and Model Design

### 2.1. Preparation of ITO Films

The K9 glass substrates were cleaned before depositing the ITO films. Then, the 80 nm ITO films were grown on cleaned K9 glasses via electron beam (EB) evaporation using ITO target materials (10 wt% SnO_2_: 90 wt% In_2_O_3_, 99%). The EB experiment was performed in less than 5.0 × 10^−4^ pa at 370 °C. In addition, when EB evaporation was carried out, 60 Sccm of oxygen was passed in, and the pressure at this time was about 5.0 × 10^−2^ pa. The humidity requirement of the air was less than 30%. To guarantee homogeneity, the deposition rate control was at 0.05 nm/s, and the substrates were rotated at 30 rpm and placed on the fixture within the same radius of the circle.

### 2.2. Synthesis of Au@Ag Nanoparticles

First, the Au nanoparticles were prepared based on the reduction in HAuCl_4_ sodium citrate according to the description by Frens G. [16]. In other words, 2.5 mL (1 wt%) HAuCl_4_ solution was diluted in 250 mL of deionized water, stirred, and heated to boiling. Meanwhile, a 2.5 mL sodium citrate solution was quickly added to the boiling mixture for 30 min. Then, heating was stopped but stirring continued until the mixture cooled down to room temperature. The prepared Au nanoparticles were centrifuged at 9000 rpm for 20 min and dispersed in 250 mL of deionized water. The dispersion solution of 250 mL of Au nanoparticles was heated and stirred to 60 °C. Then, 6 mL of sodium citrate (1 wt%) and 2.5 mL of silver nitrate solution were added. After stirring and heating for 40 min, the heating of Au@Ag solution was stopped, but stirring continued until it cooled down to room temperature. The Au@Ag nanoparticles were obtained by centrifugation at 9000 rpm for 20 min and then kept at 4 °C for further use.

### 2.3. Synthesis of ITO/Au@Ag Composites

First, the uniform 80 nm ITO films were placed on top of the experimental platform. Second, different volumes of Au@Ag nanoparticle solutions were added dropwise to the ITO films’ surface using a pipette. To ensure repeatability, the solution was added drop by drop to the surface of the ITO films until the solution was completely dried, and then the ITO/Au@Ag bimetallic nanoparticles were prepared.

**Figure 1:**
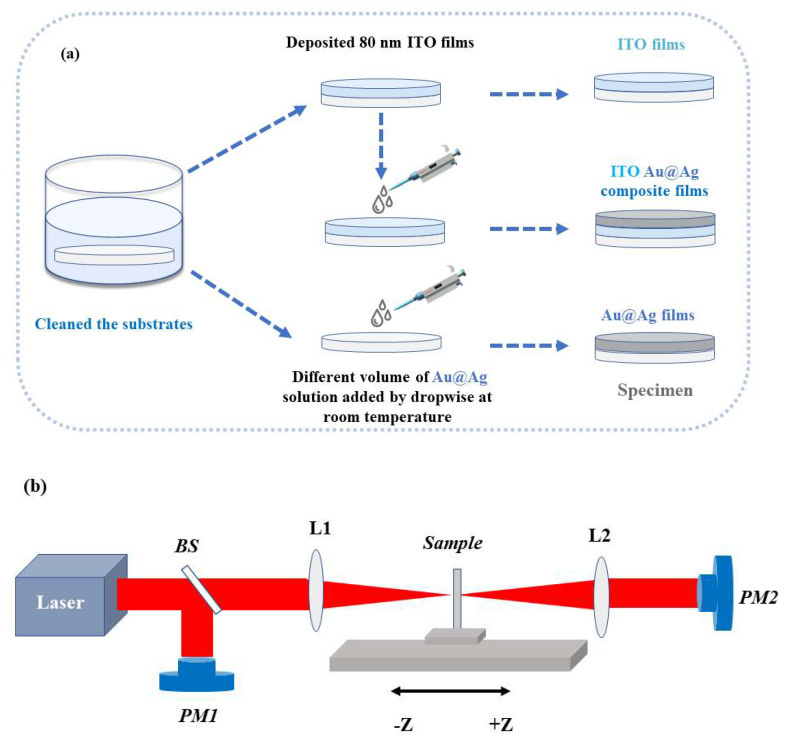
(**a**) Experimental setup and (**b**) the nonlinear open-aperture Z-scan. BS, beam splitter; PM, optical power meter; L, the convex lens.

To facilitate the comparison, ITO thin films and Au@Ag on bare substrates were also prepared under the same experimental conditions. The ITO thin film surfaces covered with 0, 20, 50, 80, 110, 140, 170, and 200 μL of Au@Ag colloids were denoted as S0, S1, S2, S3, S4, S5, S6, and S7, respectively. The K9 glasses and the K9 glasses spin-coated with Au@Ag nanoparticles were represented as K9 and Au@Ag, respectively. To better explain the preparation process, we drew the preparation flowchart as shown in Figure 1a.

The object phase information of the films was carried out through XRD analysis with Cu Kα radiation (λ = 0.15408 nm). The surface shape dimensions and surface roughness of the specimens were investigated using AFM and SEM. Linear optical spectra of the specimens were obtained via a dual-beam UV-VIS-NIR spectrophotometer (Lambda 1050, Perkins Elmer, Waltham, MA, USA). Permittivity characteristics were obtained using spectroscopic ellipsometry (Horiba, Fukuoka, Japan). The third-order NLO properties of this series of ITO/Au@Ag composite materials were investigated. We used an open-aperture Z-scan technique to characterize the nonlinear optical properties. The light source in the Z-scan device is a laser (Menlo systems, 1 MHz, 80 fs) with an output wavelength of 1550 nm (as shown in Figure 1b). The movement of the sample was performed using a stepper motor, and data acquisition was obtained using a computer to avoid human error. The experimental specimen characterization was carried out at room temperature.

## 3. Results and Analysis Discussion

**Figure 2:**
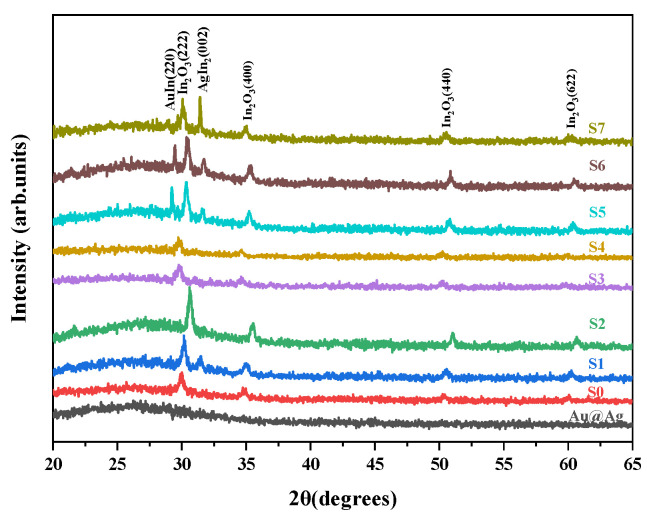
XRD patterns of the Au@Ag, ITO and ITO composites with different thicknesses of Au@Ag.

The XRD spectrum reveals the effects of Au@Ag on the structure of the ITO films, as shown in Figure 2. As shown in Figure 2, good crystalline was observed in the ITO films, which was in line with the cubic structure of In_2_O_3_ (JCPDS: 65-3170). The strongest diffraction peak at around 30.375° (2θ) corresponded to the (222) crystallographic plane. This indicates that the ITO grains are preferentially oriented along the (222) crystallographic direction. For the single Au@Ag layer in our experiment, no obvious diffraction peak with a high signal ratio was observed, indicating that the prepared single Au@Ag layer has no long-range ordered crystal structure. The diffraction peaks of the ITO Au@Ag hybrid films were different and varied with the Au@Ag thickness. When only a small amount of Au@Ag covered the ITO film surface, the intensity of the (222) diffraction peak for ITO-based films increased under the synergistic effect [17] with Au@Ag bimetallic nanoparticles. When 80 μL of Au@Ag colloid was added, the proportion of amorphous Au@Ag colloid increased; that is, the thickness of Au@Ag in the composite sample increased, which reduced the crystallinity of the ITO films. This phenomenon indicates that there is not only a synergistic effect but also competition between the ITO films and the Au@Ag layer. With the thickness of the Au@Ag layer further increasing, the (002) diffraction peaks of Agln_2_ (JCPDS: 65-1552) and (220) gold indium (JCPDS: 65-9726) were observed in the ITO/Au@Ag composite materials. At the same time, the intensity of the ITO diffraction peak in composite films also improved to a certain extent. This shows that there is an extremely strong interaction between the ITO and Au@Ag bimetallic nanoparticles, which is due to the electric field enhancement induced by the ENZ mode and LSPR [18,19]. In addition, we found that the introduction of Au@Ag not only affects the change in the intensity of the diffraction peaks of ITO films but also shifts the position of the diffraction peaks as a result of residual stress release. The XRD results show that we successfully prepared the ITO/Au@Ag composite materials.

**Figure 3:**
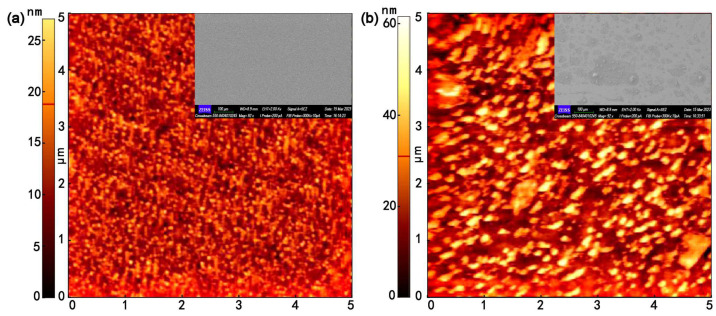
SEM and AFM micrographs of ITO films without Au@Ag layer (S3) (**a**) and with Au@Ag layer (S3) (**b**).

Figure 3a shows the AFM and SEM micrographs of the as-deposited ITO films. The value of root-mean-square surface roughness (Rq) was 3.471 nm for the as-deposited ITO films, indicating that the surface was continuous with smooth surfaces. The values of Rq increased with an increase in the Au@Ag layer on the ITO surface (as shown in Figure 3b). The Rq value of S3 was 9.326 The inserts in Figure 3a,b show the corresponding SEM images. From the SEM images, it can be seen that the as-deposited ITO films were uniformly distributed and grew orderly. When the Au@Ag nanoparticles were covered with ITO films, Au@Ag nanoparticles became dispersed on the surface of the ITO films, and occasionally, larger Au@Ag nanoparticles were present. The randomly dispersed Au@Ag nanoparticles increased the surface roughness of the ITO films.

**Figure 4:**
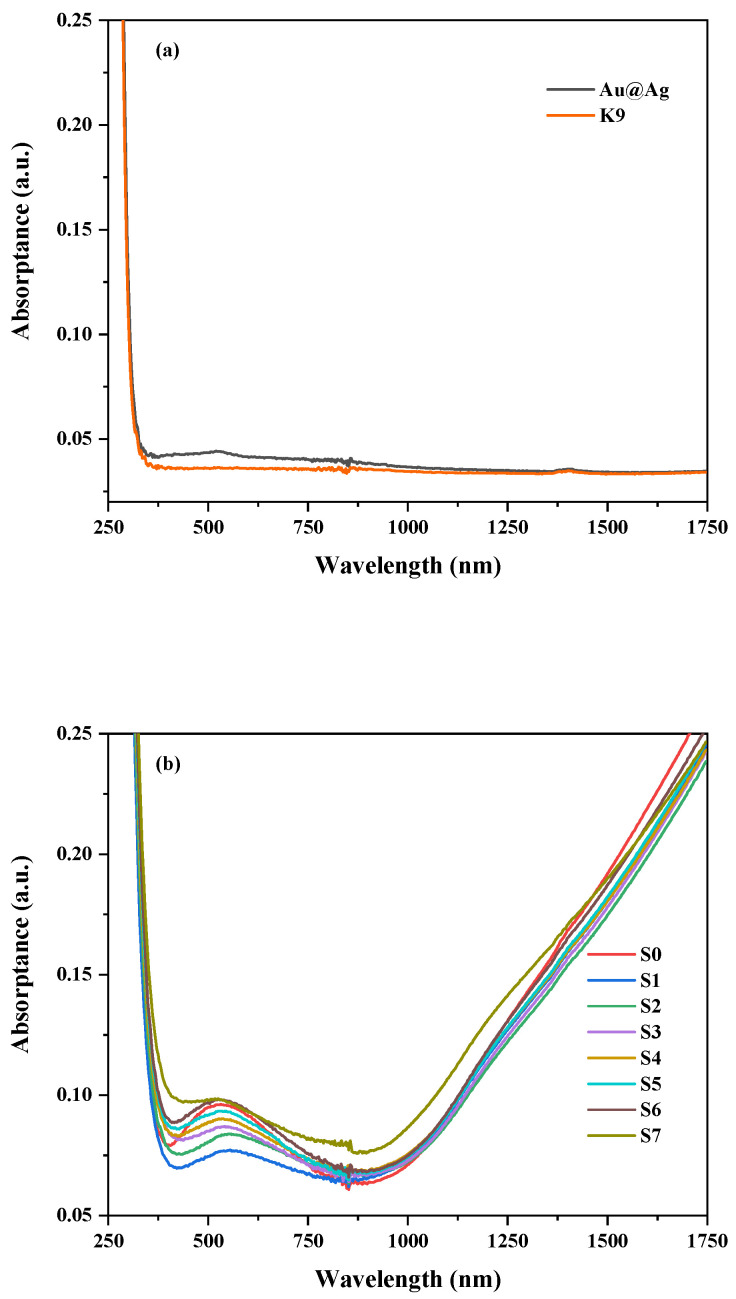
(**a**) The absorption spectra of K9 substrate and Au@Ag layer. (**b**) ITO films with various Au@Ag thicknesses.

Figure 4a shows the absorption spectrum of the k9 glasses and the Au@Ag layer. We found that the Au@Ag film sample had a peak pack near 528 nm, corresponding to the Au@Ag plasmon resonance absorption peak [20]. Since we added very little Au@Ag colloidal solution, its absorption was only about 0.05, which was at its highest when it was completely dried on the k9 substrate. Figure 4b shows that the absorption spectrum of the ITO films varied with the Au@Ag thicknesses. As shown in Figure 4b, the S0 sample has an interference peak in the visible region and a broad plasmon resonance peak in the near-infrared (NIR) region. The variation in the absorption peak of ITO/Au@Ag composites in the visible region is mainly caused by the LSPR of Au@Ag bimetallic nanoparticles. As shown in Figure 4b, with increasing thickness of the Au@Ag, the intensity of the plasmon resonance absorption peak of the composite material increases gradually in the visible region, and the resonance peak position also shifts range from 528 to 560 nm. Since the ITO ENZ region is located in the NIR region [2], the electric field enhancement effect is mainly provided by the ENZ mode [18] when a small amount of Au@Ag covering the surface of the ITO films does not have a significant effect on the NIR absorption of composite films. In the S7 sample, when the amount of Au@Ag reached 200 μL, a surface plasmon resonance absorption peak was observed at about 1200 nm in the NIR region, indicating that there is a strong interaction [21,22] at the interface of the ITO and Au@Ag bimetallic nanoparticles.

**Figure 5:**
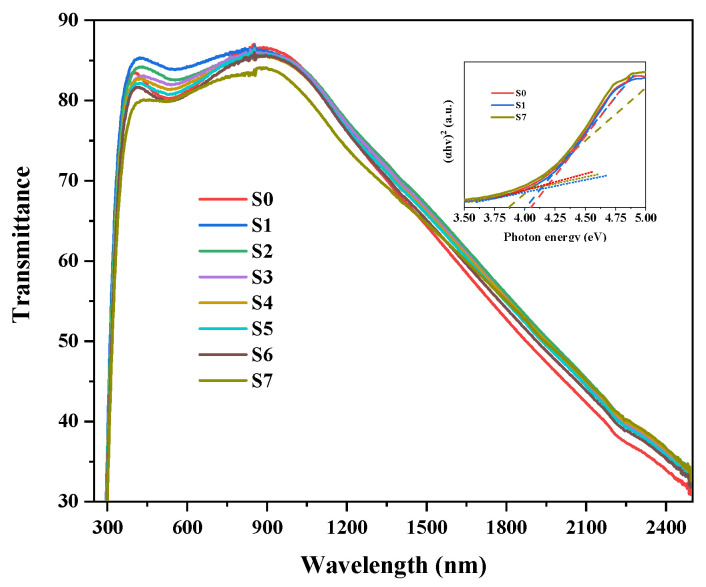
Transmittance spectrum for ITO films with different thicknesses of Au@Ag. The inset shows the optical band gaps of samples S0, S1, and S7. The dash in the insertion is the fitted line and the short dot is the baseline.

Figure 5 represents the transmittance curves of the ITO and ITO/Au@Ag composite films. The insertion shows the optical band gaps of samples S0, S1, and S7. As shown in Figure 5, the transmittance of S0 is greater than 80% in the visible region. With the increasing thickness of Au@Ag, the transmittance values of ITO/Au@Ag decreased gradually as a result of the absorption of Au@Ag nanoparticles. To study the effects of Au@Ag introduction on the band gap of ITO films, we calculated the values of 4.13 (S0), 4.05 (S1), and 3.95 eV (S7) samples (inserted in Figure 5) by following the proposed method for correct calculation band gap by Patrycja Makuła et al. [23]. The variation in the band gap indicated that the introduction of Au@Ag nanoparticles remarkably reduced the bandgap energy because of their localized surface plasmon resonance effects [24].

**Figure 6:**
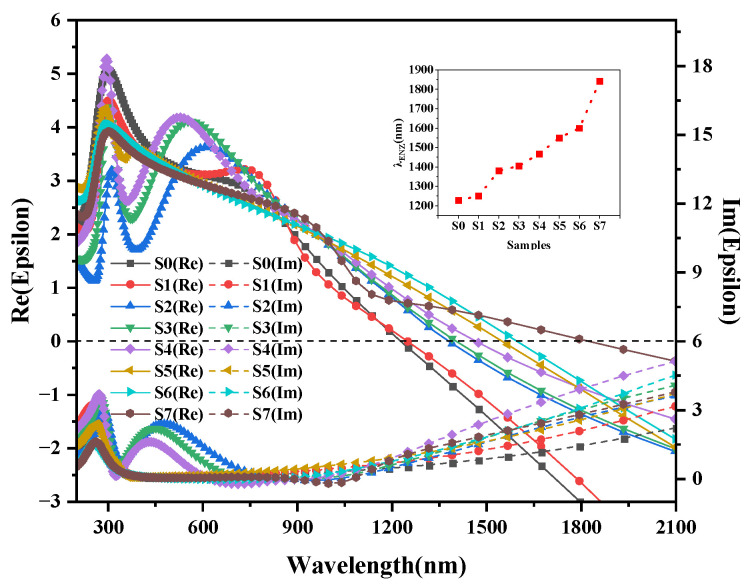
The permittivity of ITO films and ITO/Au@Ag composites with real (solid line) and imaginary (dash line) parts. The inset includes the λ_ENZ_ of the specimen.

Figure 6 shows the measured permittivity of ITO and ITO with various Au@Ag thicknesses retrieved from the spectral ellipsometry and fitted by the Drude–Lorentz model [25,26] and effective medium approximation [27]. For the spectral ellipsometry modeling, we used DeltaPsi 2 software (HoRIBA, Jobin). From bottom to top, the model included (i) the void layer, (ii) K9 glass, (iii) the ITO film, and (iv) Au@Ag nanoparticles. When using the effective medium approximation model to fit the spectral ellipsometry data, the ITO films are used as the main body and Au@Ag as the component. The mean-square-error values obtained by matching the spectroscopic ellipsometry data were below 5 for ITO matrix composites. As can be seen from the comparisons of ITO films with and without Au@Ag nanoparticles in Figure 6, the introduction of Au@Ag shifts the real permittivity curves. We found that the ENZ real part wavelength of the ITO films varied from 1228 nm (S0) to 1843 nm (S7), with only a small amount of alternation of the Au@Ag content. Although the introduction of Au@Ag nanoparticles seems to bring in free electrons, there are more interfaces between the ITO films and the larger Au@Ag bimetallic nanoparticles. These interfaces increase the scattering and block out carriers [27], which reduces the effective carrier concentrations. The plasmon resonance frequency became smaller with the decrease in the effective carrier concentration; that is, the real part of the ENZ moved in the direction of the long wave. The imaginary part of permittivity increased by a relatively reasonable amount. Small changes in the dielectric constant can induce large nonlinear optical properties. Moreover, the crystalline status of the ITO films is highly susceptible to the random dispersion of innumerable Au@Ag particles, which in turn affects the intensity of the ITO diffraction peaks (Figure 2).

In order to investigate the NLO properties of the specimens, we measured the nonlinear absorption coefficient using the OA Z-scan experimental setup (Figure 1b). In the Z-scan experimental setup, we used a 1550 nm laser (80 fs, 1 MHz) as an excitation source with an intensity of 282.9 GW/cm^2^. The movement of the sample was performed using a stepper motor with a step size of 0.5 mm and the optical power variation data were collected automatically by a computer. The laser beam exited from the laser and passed through a beam splitter to split into two beams. One of the laser beams was used as a detection light to characterize the nonlinear absorption of the ITO/Au@Ag specimen prepared by us, and the other beam was used as a reference light to eliminate the effects of the laser’s own fluctuations. The NLO response of the sample was stimulated by a drastic change in the light intensity near the focal point (*Z* = 0). We used an optical power meter to collect the transmittance of the ITO composites at different locations. The nonlinear absorption coefficients of ITO with and without the Au@Ag layer were calculated and analyzed. The NLO parameters were extracted using the OA Z-scan theory and model fitting results. The total absorption of the ITO/Au@Ag composite films can be expressed by the following formula:

(1) $$\alpha (I)=\alpha_{0}\frac{1}{1+I_{0}/I_{s}}+\beta I$$

Here, *α*_0_ represents the linear absorption coefficient, *I*_0_ represents laser beam peak intensity, *I_s_* represents saturation intensity, and *β* is the nonlinear absorption coefficient.

The normalized transmittance as a relation of specimen position *z* can be obtained by the following formula [28]:

(2) $$T(z)=\frac{1}{\sqrt{\pi }q_{0}}\int_{-\infty }^{\infty }ln\left[1+q_{0}exp(-{\left(\frac{z}{z_{0}}\right)}^{2})\right]d(\frac{z}{z_{0}})$$

where T(z) is the normalized transmittance, *Z* is the relative focus position of the sample, $Z_{0}=\frac{kw_{0}^{2}}{2}$ is the Rayleigh length, *k* is the wavenumber, $w_{0}$ is the beam radius at the 1/e^2^ level of the intensity distribution, and $q_{0}=\beta I_{0}L_{eff}$, *I*_0_ is the intensity for an on-axis laser beam; $L_{eff}=\frac{1-\exp(-\alpha L)}{\alpha }$ represents the effective interaction length, and *L* is the actual thickness of the samples.

The phenomenon of the two-photon absorption and RSA can be expressed well by the proposed theoretical model of Sheik-Bahae and Chapple [29,30], as shown below:

(3) $$T_{RSA}(z)=(1+\frac{\alpha_{0}}{1+\frac{I}{I_{sat}}})\times (1-\frac{\beta I_{0}L_{eff}}{2\sqrt{2}(1+{\left(\frac{z}{z_{0}}\right)}^{2})})$$

Additionally, the SA can be described well by Rao et al. [31] as follows:

(4) $$T_{SA}(z)=1+\frac{I_{0}}{I_{sat}(1+{\left(\frac{z}{z_{0}}\right)}^{2})}$$

where $I_{sat}$ represents the saturation intensity of the materials, *Z* is the relative focus position of the sample, $Z_{0}=\frac{kw_{0}^{2}}{2}$ is the Rayleigh length, *k* is the wavenumber, and $w_{0}$ is the beam radius at the 1/e^2^ level of the intensity distribution.

**Figure 7:**
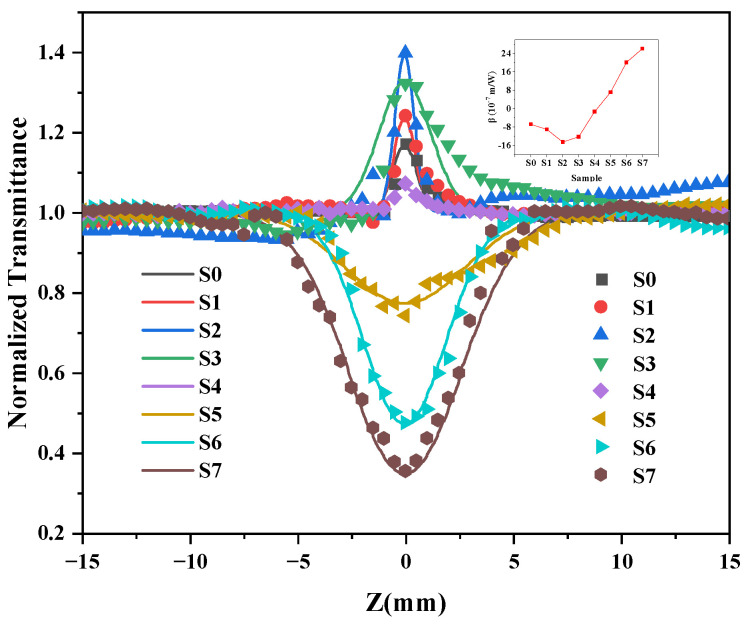
The OA Z-scan results of ITO films with various Au@Ag thicknesses. The inset shows the change of nonlinear saturation absorption coefficient of S0 to S7.

The normalized transmittance curve for the ITO films and ITO films with various thicknesses of Au@Ag nanoparticles is shown in Figure 7. The solid line in Figure 7 is the normalized fitted data, and the square dots are the measured raw data. The inset of Figure 7 shows the nonlinear absorption coefficients of the samples numbered S0–S7. It should be explicitly pointed out that the prepared Au@Ag nanoparticles were randomly dispersed, and the Au@Ag on the bare substrate did not show symmetrical and regular characteristics on either side of the focus point in the Z-scan test. It can be seen in Figure 7 and in the insert images that the normalized transmittance curve for the ITO films shows a symmetrical peak shape on both sides of the focus (*Z* = 0). The β of ITO films is −6.85 × 10^−7^ m/W. Under the same input intensity as that of the ITO films, the ITO films with various Au@Ag thicknesses transitioned from SA to RSA. The nonlinear saturable absorption coefficient of ITO/Au@Ag composites ranges from −9.08 × 10^−7^ to 26.06 × 10^−7^ m/W. In addition, when the composite specimen presents an SA behavior, its normalized transmittance first increases and then decreases. This is mainly because of the dominant ratio of ITO films in the composite specimens. A small amount of Au@Ag enhances the NLO response of the composite specimen under the effect of localized surface plasmon resonance [32]. As the proportion of Au@Ag in the composite specimens increases, there are more interfaces between the random dispersion of larger Au@Ag bimetallic nanoparticles and ITO films, which block the carrier movement and increase the scatter. Therefore, the normalized transmittance of the composite specimens decreased gradually. With a further increase in the Au@Ag content, Au@Ag plays a dominant role in the composite specimen, and the total absorption of the composite specimen increases, showing an RSA behavior. This is primarily because, for non-resonant excitation, when the excited state absorption cross-section is larger than that of the ground state absorption, the nonlinear absorption behavior is dominated by two-photon absorption and RSA [33,34]. Based on this, ITO/Au@Ag composites have great potential for optical switching, optical limiting, and many other nonlinear applications.

**Figure 8:**
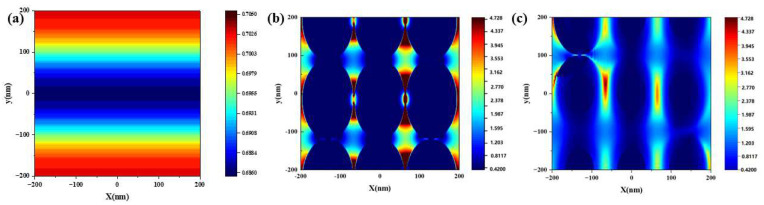
FDTD simulation patterns of (**a**) ITO films, (**b**) Au@Ag films, and (**c**) ITO/Au@Ag composite films.

The electric field variation for Au@Ag films, ITO films, and ITO film surfaces covered with Au@Ag films was calculated by FDTD to verify the above conclusion (as shown in Figure 8). In the x-y plane, we selected a rectangle of −200–200 nm as a representative region for the simulation of the periodic structure. The bottom was the substrate (SiO_2_) with a thickness of 1.35 mm and the middle layer was the ENZ (ITO films, 80 nm). The top layer was Au@Ag with different thicknesses. Perpendicular to the x-y plane of the specimen, we used a 532 nm laser for irradiation. The polarization was along the y-axis direction. The parameters used in the simulation were the same as those used for the experimental test data. The distribution of the electric field for the single-layer ITO films was dense and uniform, and the intensity was only about 0.705. Figure 8b,c represent the electric field distribution at the junction between Au@Ag and the buffer layer (bare substrate and ITO films), respectively. The maximum joint electric field intensity enhancement factors of Au@Ag were larger than that of ITO/Au@Ag, which can be attributed to the EM resonance excitation of the localized surface plasmon resonance. In general, the calculated results agree well with the experimental results.

## 4. Conclusions

In summary, we prepared aqueous suspensions of Au@Ag using a chemical reduction method. We investigated the effects of Au@Ag nanoparticles on the optical properties, structure, and morphology of ITO films in this paper. The Au@Ag nanoparticles deposited on the surface of the ITO films (K9 glasses) were characterized using a UV-Vis-NIR spectrophotometer and XRD. The nonlinear optical properties were determined using an OA Z-scan regime pumped at 1550 nm with a pulse duration of 80 fs. We observed that it is possible to make a more efficient control of NLO with the construction of hybrid semiconductors with metallic nanostructures because it is possible to control the surface plasmon resonance by controlling the amount of Au@Ag. By increasing the Au@Ag thickness, the wavelength range of the real part of ENZ could be adjusted from 1228 to 1843 nm. In this ITO matrix composite, a transition from SA to RSA was observed. Furthermore, the results indicate that the tunable NLO properties realized can be attributed to the strong interaction between the ITO film and Au@Ag nanoparticles based on the ENZ mode and localized surface plasmon resonance. Thus, we can present ITO/Au@Ag composite materials as good candidates for applications in photonics (optical switching and optical limitation).

## Data Availability

All the data supporting reported resulted results can be found in the text of this paper.

## References

[1] Autere A., Jussila H., Dai Y., Wang Y., Lipsanen H., Sun Z. (2018). Nonlinear Optics with 2D Layered Materials. Adv. Mater..

[2] Alam M.Z., De Leon I., Boyd R.W. (2016). Large optical nonlinearity of indium tin oxide in its epsilon-near-zero region. Science.

[3] Zhang F., Xiao X., Lu Y., Dong J., Chen Y. (2023). Broadband Enhancement of Optical Nonlinearity in a Plasmonic Nanocavity Coupled with an Epsilon-Near-Zero Film. J. Phys. Chem. C.

[4] Abhijith T., Edappadikkunnummal S., Suthar R., Thomas S., Karak S. (2023). Au–WS2 Nanohybrids with Enhanced Optical Nonlinearity for Optical Limiting Applications. ACS Appl. Nano Mater..

[5] Prabhakar Vattikuti S.V., Nagajyothi P.C., Devarayapalli K.C., Yoo K., Dang Nam N., Shim J. (2020). Hybrid Ag/MoS2 nanosheets for efficient electrocatalytic oxygen reduction. Appl. Surf. Sci..

[6] Yang X., Yu H., Guo X., Ding Q., Pullerits T., Wang R., Zhang G., Liang W., Sun M. (2017). Plasmon-exciton coupling of monolayer MoS2-Ag nanoparticles hybrids for surface catalytic reaction. Mater. Today Energy.

[7] Pan R., Kang J., Li Y., Zhang Z., Li R., Yang Y. (2022). Highly Enhanced Photoluminescence of Monolayer MoS2 in Plasmonic Hybrids with Double-Layer Stacked Ag Nanoparticles. ACS Appl. Mater. Interfaces.

[8] Jiang R., Li B., Fang C., Wang J. (2014). Metal/Semiconductor Hybrid Nanostructures for Plasmon-Enhanced Applications. Adv. Mater..

[9] Guo P., Schaller R.D., Ocola L.E., Diroll B.T., Ketterson J.B., Chang R.P. (2016). Large optical nonlinearity of ITO nanorods for sub-picosecond all-optical modulation of the full-visible spectrum. Nat. Commun..

[10] Ma H., Zhao Y., Shao Y., Lian Y., Zhang W., Hu G., Leng Y., Shao J. (2021). Principles to tailor the saturable and reverse saturable absorption of epsilon-near-zero material. Photonics Res..

[11] Lau K.Y., Yang Y.T., Zhao D., Liu X.F., Qiu J.R. (2022). Tunable optical nonlinearity of indium tin oxide for optical switching in epsilon-near-zero region. Nanophotonics.

[12] Edappadikkunnummal S., Nherakkayyil S.N., Kuttippurath V., Chalil D.M., Desai N.R., Keloth C. (2017). Surface Plasmon Assisted Enhancement in the Nonlinear Optical Properties of Phenothiazine by Gold Nanoparticle. J. Phys. Chem. C.

[13] Lee K.-S., El-Sayed M.A. (2006). Gold and Silver Nanoparticles in Sensing and Imaging:  Sensitivity of Plasmon Response to Size, Shape, and Metal Composition. J. Phys. Chem. B.

[14] Elim H.I., Ji W., Yang J., Lee J.Y. (2008). Intensity-dependent enhancement of saturable absorption in PbS–Au4 nanohybrid composites: Evidence for resonant energy transfer by Auger recombination. Appl. Phys. Lett..

[15] Shiju E., Abhijith T., Chandrasekharan K. (2021). Nonlinear optical behavior of Au@Ag core-shell nanostructures. J. Mol. Liq..

[16] Frens G. (1973). Controlled Nucleation for the Regulation of the Particle Size in Monodisperse Gold Suspensions. Nat. Phys. Sci..

[17] Shan H., He J., Zhu B., Zhou J., Huo H. (2023). Synergistic effects of ultrasonication and the addition of a nucleating agent on improving the crystallization and performance of P3HT. Org. Electron..

[18] Jiang H., Zhao Y., Ma H., Feng C., Wu Y., Zhang W., Chen M., Wang M., Lian Y., Cao Z. (2022). Polarization-Independent, tunable, broadband perfect absorber based on semi-sphere patterned Epsilon-Near-Zero films. Appl. Surf. Sci..

[19] Lian J., Zhang D., Hong R., Yan T., Lv T., Zhang D. (2019). Broadband Absorption Tailoring of SiO_2_/Cu/ITO Arrays Based on Hybrid Coupled Resonance Mode. Nanomaterials.

[20] Wu G.-F., Zhu J., Weng G.-J., Cai H.-Y., Li J.-J., Zhao J.-W. (2023). The structure and plasmonic properties regulation of Au@Ag core-shell nanostructures with Au triangular nanoprisms as the core mediated by halide. J. Alloys Compd..

[21] Ma Y., Li Q., Wang S., Wang Y., Liu H., Wang X., Zhao B., Jiang Z., Ruan W. (2022). Observation of tunable surface plasmon resonances and surface enhanced infrared absorption (SEIRA) based on indium tin oxide (ITO) nanoparticle substrates. Spectrochim. Acta Part A Mol. Biomol. Spectrosc..

[22] Dai Y., Yan T., Hong R., Tao C., Lin H., Wang Q., Zhang D. (2021). Graphene oxide induced the enhancement of nonlinear optical response of ITO films. Opt. Mater..

[23] Makuła P., Pacia M., Macyk W. (2018). How To Correctly Determine the Band Gap Energy of Modified Semiconductor Photocatalysts Based on UV–Vis Spectra. J. Phys. Chem. Lett..

[24] Zhang W., Li Q., Xia H. (2023). Photocatalytic oxidation of 5-hydroxymethylfurfural to furandicarboxylic acid over the Au-Ag/TiO_2_ catalysts under visible light irradiation. Appl. Surf. Sci..

[25] Guo P., Chang R.P.H., Schaller R.D. (2017). Transient Negative Optical Nonlinearity of Indium Oxide Nanorod Arrays in the Full-Visible Range. ACS Photonics.

[26] Shi K., Haque R.R., Zhao B., Zhao R., Lu Z. (2014). Broadband electro-optical modulator based on transparent conducting oxide. Opt. Lett..

[27] Chen C., Wang Z., Wu K., Ye H. (2018). Tunable near-infrared epsilon-near-zero and plasmonic properties of Ag-ITO co-sputtered composite films. Sci. Technol. Adv. Mater..

[28] Gu B., He J., Ji W., Wang H.-T. (2008). Three-photon absorption saturation in ZnO and ZnS crystals. J. Appl. Phys..

[29] Sheik-Bahae M., Said A.A., Wei T.H., Hagan D.J., Stryland E.W.V. (1990). Sensitive measurement of optical nonlinearities using a single beam. IEEE J. Quantum Electron..

[30] Chapple P.B., Staromlynska J., Hermann J.A., McKay T.J., McDuff R.G. (1997). Single-Beam Z-Scan: Measurement Techniques and Analysis. J. Nonlinear Opt. Phys. Mater..

[31] Rao K.S., Ganeev R.A., Zhang K., Fu Y., Boltaev G.S., Krishnendu P.S., Redkin P.V., Guo C. (2018). Laser ablation–induced synthesis and nonlinear optical characterization of titanium and cobalt nanoparticles. J. Nanoparticle Res..

[32] Chen Y., Lu Z., Cao Y., Sun M., Dong J. (2022). Polarization and incident angle-dependent plasmonic coupling of Au@Ag nanoalloys. Chin. J. Phys..

[33] Liu Z., Wang Y., Zhang X., Xu Y., Chen Y., Tian J. (2009). Nonlinear optical properties of graphene oxide in nanosecond and picosecond regimes. Appl. Phys. Lett..

[34] Yan T., Hong R., Tao C., Wang Q., Lin H., Han Z., Zhang D. (2022). Thickness dependency of PVA on the transition from saturable absorption to reverse saturable absorption of ITO films. Opt. Mater..

